# Correction: Raha et al. Lipid-Lowering Drug Gemfibrozil Protects Mice from Tay-Sachs Disease via Peroxisome Proliferator-Activated Receptor α. *Cells* 2023, *12*, 2791

**DOI:** 10.3390/cells13161374

**Published:** 2024-08-19

**Authors:** Sumita Raha, Debashis Dutta, Ramesh K. Paidi, Kalipada Pahan

**Affiliations:** 1 Department of Neurological Sciences, Rush University Medical Center, Chicago, IL 60612, USA; sumitaraha@gmail.com (S.R.); duttad@musc.edu (D.D.); ramesh_kumar_paidi@rush.edu (R.K.P.); 2 Division of Research and Development, Jesse Brown Veterans Affairs Medical Center, 820 South Damen Avenue, Chicago, IL 60612, USA

**Error in Figure 4**

In the original publication [1], there was a mistake in Figure 4. One image was incorrectly provided for the Non-Tg (upper left panel) group of Figure 4E that had an overlap with Figure 4C—PAS. This happened due to the simultaneous processing of numerous images, involving contrasting and cropping, while working with multiple open windows on a laptop. It has now been corrected. Please see the corrected Figure 4E below.

**Figure 4:**
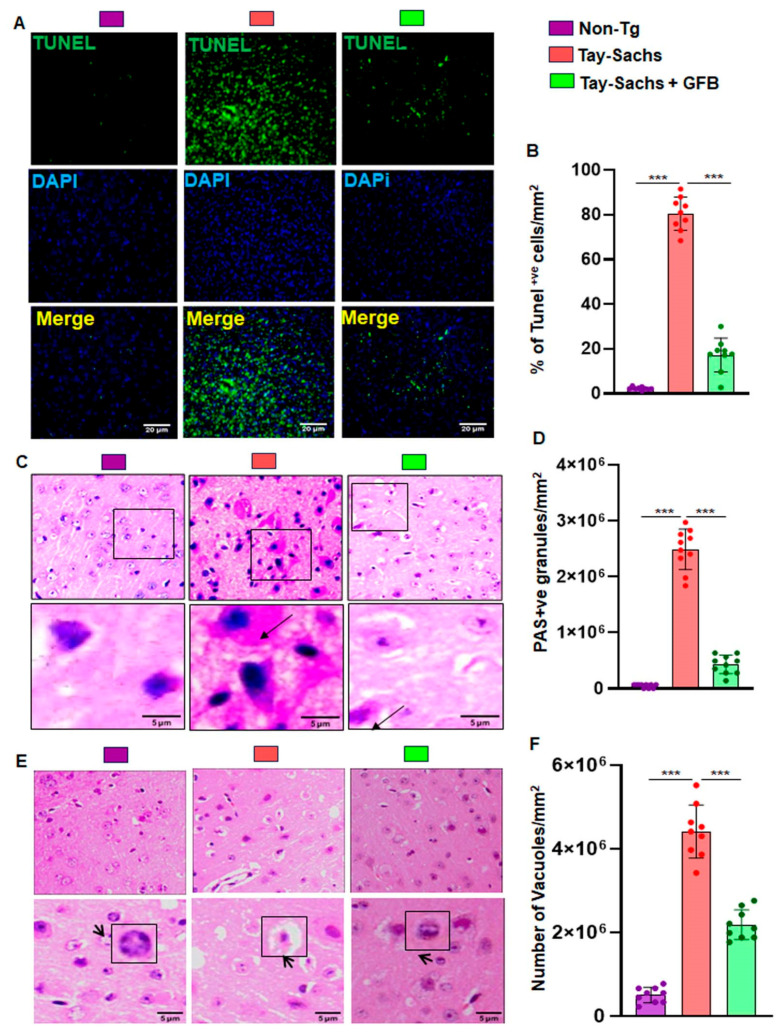
Treatment with GFB attenuates neuronal apoptosis and reduces glycoconjugates in Tay-Sachs mice. Three-month-old TS mice (*n* = 6/group) were orally administered with GFB (8 mg/kg/d) solubilized in 0.5% methylcellulose. Therefore, control TS mice received 0.5% methylcellulose as a vehicle. After 2 months of treatment, cortical sections were stained for TUNEL (**A**). TUNEL (+ve) cells were expressed as a % of total cells per square mm (**B**). Paraffin-embedded cerebral cortex sections were subjected to periodic acid-Schiff (PAS) stain for analyzing glycolipids (**C**). The glycoconjugate material-stained magenta is shown by the arrow and quantified for PAS +ve cells per mm square (**D**). Paraffin-embedded sections were also subjected to H & E staining as characterized by large vacuoles (thick arrows), pyknotic nuclei and swollen neuron in TS mice (**E**). The number of vacuoles was quantified per mm square (**F**). Results represent counting three different sections from 3 different mice (*n* = 3) per group. All data represent mean ± SEM. All statistical analysis was performed by one-way ANOVA, followed by Dunnett’s multiple comparison test. *** *p* < 0.001.

**Wrong Model**

We would also like to report an error in the strain of the animals that was said to have been used in this study. Throughout the study, we used B6;129S4-Hexb^tm1Rlp^/J mice lacking the *Hexb* gene, which model a severe form of Tay–Sachs (Sandhoff) disease.

The authors state that the scientific conclusions are unaffected. This correction was approved by the Academic Editor. The original publication has also been updated.

## References

[1] Raha S., Dutta D., Paidi R.K., Pahan K. (2023). Lipid-Lowering Drug Gemfibrozil Protects Mice from Tay-Sachs Disease via Peroxisome Proliferator-Activated Receptor α. Cells.

